# Isolation, Biosynthesis, and Biological Activity of Polycyclic Xanthones From Actinomycetes

**DOI:** 10.3389/fmicb.2022.922089

**Published:** 2022-07-13

**Authors:** Hui-Qing Yu, Gang Li, Hong-Xiang Lou

**Affiliations:** ^1^School of Basic Medicine, Qingdao University, Qingdao, China; ^2^Department of Natural Medicinal Chemistry and Pharmacognosy, School of Pharmacy, Qingdao University, Qingdao, China; ^3^Department of Natural Product Chemistry, Key Laboratory of Chemical Biology of Ministry of Education, School of Pharmaceutical Sciences, Shandong University, Jinan, China

**Keywords:** actinomycetes, natural products, polycyclic xanthones, biosynthesis, bioactivities

## Abstract

Natural products from actinomycetes serve as a crucial source of clinical pharmaceuticals, especially antibiotics and anticancer agents. Among them, polycyclic xanthones belong to a growing group of highly oxygenated aromatic polyketides with a xanthone-containing angular hexacyclic framework. These biosynthetically unique small molecules are of great interest due to their wide spectrum of biological activities, especially the remarkable antibacterial activity against gram-positive bacteria and the significant antineoplastic effects toward various cancer cells at nanomolar concentrations. Their complex structures and significant bioactivities have aroused considerable attention in the chemical and biological communities in recent decades. This review covers the isolation, the biosynthesis, and the biological studies toward these structurally complex and biologically active molecules.

## Introduction

Natural products with enormous scaffold diversity and structural complexity play a fundamental role in the drug discovery pipeline (Newman and Cragg, 2020; Atanasov et al., 2021). These molecules have various modes of action and can be used directly or employed as leads for optimization into new drugs (Li and Lou, 2018). Actinomycetes, as gram-positive bacteria distributed in both terrestrial and marine ecosystems, have been recognized as one of the most prolific sources of structurally diverse and biologically active natural products (Genilloud, 2017; Jagannathan et al., 2021). Many success stories to emerge from these actinomycetes-derived novel secondary metabolites as drugs are noteworthy (Jose et al., 2021). One of them is the Waksman’s rational screening, isolation, and clinical approval of the drug streptomycin, the first effective treatment for tuberculosis (Waksman, 1953). The discovery and development of anticancer antibiotics exemplified by anthracyclines, are also particularly intriguing in recent decades (Gao et al., 2020). However, since the 1980s, the discovery of novel natural products from actinomycetes was significantly hindered by the re-isolation of known compounds, which was further terribly coincided with a growing emergence of drug resistance (Behie et al., 2017). To address this problem, attention is turning to some “old compounds” with unique structural pharmacophores and significant bioactivity (Tian et al., 2022).

**FIGURE 1:**
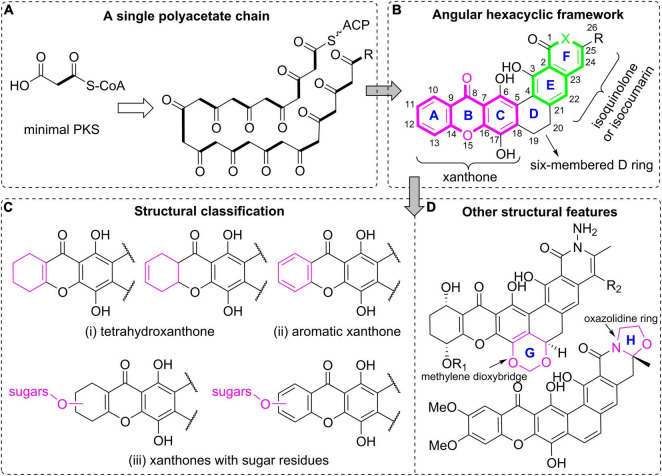
Polycyclic xanthone natural products. **(A)** A single polyacetate chain as the biosynthetic precursor; **(B)** the general polycyclic xanthone structure with an angular hexacyclic framework; **(C)** structural classification based on the oxidation and substitution features of the xanthone core; **(D)** other structural features for further classification.

Polycyclic xanthones (Figure 1), which belong to a small group of actinomycetes-derived aromatic polyketides, have been known for nearly 50 years (Gurevich et al., 1972; Winter et al., 2013). They are featured by an angular hexacyclic framework that is highly oxygenated and contains a xanthone substructure and a isoquinolone or isochromane moiety (Figure 1; Winter et al., 2013). In this family, the polycyclic skeleton including the specific xanthone unit originated from a single polyacetate chain, is assembled by a type II polyketide synthase (PKS) (Hertweck et al., 2007; Tolmie et al., 2019). The last decades have seen noticeable successes in isolation and biosynthetic studies on this intriguing class of large polycyclic compounds (Kong et al., 2020b). More importantly, this class of molecules have displayed diverse biological activities at the nanomolar range, especially their strong antimicrobial activities and the significant antineoplastic effects toward various cancer cells (Winter et al., 2013; Annang et al., 2018; Hu et al., 2020).

The unique chemical structures of polycyclic xanthones in combination with their remarkable bioactivities have driven a number of chemical, biosynthetic, and biological studies on this family. There is only one highlight article on polycyclic xanthones in 2013 (Winter et al., 2013). It mainly presented the synthetic and pharmacological advances of this group (Winter et al., 2013). However, as far as we know, detailed isolation and biosynthesis of polycyclic xanthones have not been reviewed.

Therefore, given the current gaps in a comprehensive understanding of polycyclic xanthones and considering their intriguing chemistry and biology, a number of research articles from 1972 to 2021 dealing with their isolation, biosynthesis, and biological studies are collected and summarized in this review. The aim of this review is to offer an informative overview of these research advances and to facilitate further elaboration of this old promising class of anticancer antibiotics.

## Structural Classification

Polycyclic xanthone derivatives are an expanding group of structurally complex aromatic compounds that typically originate from a single C26 or C28 polyketide chain (Figure 1A). They are represented by an angular polycyclic framework composed of a highly oxygenated xanthone core along with an isoquinolin-1(2*H*)-one or isocoumarin (Figure 1B). Their structural diversity is mainly attributed to the variations in oxidation states of the xanthone ring, and its diverse substitutions including hydroxyl groups, halogen atoms and sugar residues, together with the presence of a methylene dioxybridge or a oxazolidine ring fused to the hexacyclic skeleton, and the quinone/hydroquinone oxidation state. It is noteworthy that the molecular twist presented in the isokibdelones were also found and generated an unprecedented heterocyclic system (Ratnayake et al., 2006). In this review, those structurally complex natural products are mainly classified according to the oxidation and substitution features of the xanthone core (Winter et al., 2013). Specifically, they are broadly divided into three groups: (i) tetrahydroxanthones; (ii) aromatic xanthones; (iii) xanthones with sugar residues (Figure 1C). The compounds are further classified according to the presence of a methylene dioxybridge or a fused oxazolidine ring in the polycyclic framework (Figure 1D).

## Isolation and Identification

Many chemists focus on the chemical isolation and identification of polycyclic xanthones with the intension to discover more biologically active compounds (Winter et al., 2013). This class of secondary metabolites are increasingly being uncovered from actinomycetes and contribute to a bioactive compound library for modern drug discovery. *Streptomycetes* are the most chemically studied actinobacteria and proven to have an extraordinary ability to produce colored polycyclic xanthones (Winter et al., 2013). Currently, their fermentation broths were frequently extracted with an equal volume of ethyl acetate three times to obtain the crude extracts (Hu et al., 2019; Xu et al., 2020; Ye et al., 2020; She et al., 2021). Further isolation and identification procedure might be hardly hindered by the low solubility of this kind of pigments in many organic solvents (Nakagawa et al., 1986; Hu et al., 2020), and chemical modification by acetylation or methylation could be used as a solution to address this problem (Ömura et al., 1986). It is expected that semi-preparative high performance liquid chromatography is the mainly used technique for final purification (Hu et al., 2019; Xu et al., 2020; Ye et al., 2020; She et al., 2021). Most of their structures and stereochemistry were elucidated by extensive spectroscopic analysis using MS (Table 1), nuclear magnetic resonance (NMR), single-crystal X-ray diffraction, as well as electronic circular dichroism (ECD) calculations, together with related chemical synthesis. Specifically, their ^1^H NMR spectra provide the key information of several aromatic/olefinic protons and oxygenated methylenes or methines, as well as few exchangeable protons, while their ^13^C NMR spectra showed a number of aromatic, olefinic, or carbonyl carbons, in addition to few saturated carbon signals (Hu et al., 2019, 2020; Xu et al., 2020; She et al., 2021).

**TABLE 1:** Structurally diverse polycyclic xanthones from actinomycetes.

Compounds	Molecular formula (Molecular weight)	Source	Notable activities	References
**Tetrahydroxanthones**				

Albofungin (**1**)*^a^*	C_27_H_24_N_2_O_9_ (520.4940)	*Actinomyces* sp. *Streptomyces chrestomyceticus*	Anti-gram-positive bacterial Cytotoxicity Nematocidal	Gurevich et al., 1972; Ratnayake et al., 2007; She et al., 2021
Chloroalbofungin (**2**)	C_27_H_23_N_2_O_9_Cl (554.9360)	*Streptomyces chrestomyceticus*	Anti-gram-positive bacterial Cytotoxicity	She et al., 2021
Albofungin A (**3**)	C_26_H_22_N_2_O_9_ (506.4670)		Anti-gram-positive bacterial Gram-negative bacteria Cytotoxicity	
Albofungin B (**4**)	C_28_H_25_NO_9_ (519.5060)		Anti-gram-positive bacterial Cytotoxicity	
Chrestoxanthone A (**5**)	C_27_H_23_NO_9_ (505.4790)	*Streptomyces chrestomyceticus* BCC 24770	Antifungal	Bunyapaiboonsri et al., 2016
Chrestoxanthone B (**6**)	C_26_H_21_NO_9_ (491.4520)		Antifungal	
Actinoplanone A (**7**)	C_28_H_25_N_2_O_10_Cl (584.9620)	*Actinoplanes* sp. R-304	Anti-gram-positive bacterial Gram-negative bacteria Antifungal (*Pyricularia oryzae*) Cytotoxicity	Kobayashi et al., 1988a,b
Actinoplanone B (**8**)	C_28_H_24_NO_10_Cl (569.9470)			
Actinoplanone C (**9**)	C_28_H_26_N_2_O_10_ (550.5200)		Antifungal (*Pyricularia oryzae*) Cytotoxicity	
Actinoplanone D (**10**)	C_28_H_25_NO_10_ (535.5050)			
Actinoplanone E (**11**)	C_31_H_29_N_2_O_10_Cl (625.0270)			
Actinoplanone F (**12**)	C_32_H_29_N_2_O_11_Cl (653.0370)			
Actinoplanone G (**13**)	C_32_H_30_N_2_O_11_ (618.5950)			
MDN-0185 (**14**)	C_26_H_21_NO_10_ (507.4510)	*Micromonospora* sp. CA-256353	Antimalarial	Annang et al., 2018
Sch 54445 (**15**)	C_30_H_29_N_2_O_9_Cl (597.0170)	*Actinoplanes* sp. ATCC 55600	Antifungal Cytotoxicity	Chu et al., 1997
Actinomadurone (**16**)	C_34_H_31_NO_10_ (613.6190)	*Actinomadura* sp. BCC 35430	Antifungal Cytotoxicity	Bunyapaiboonsri et al., 2017
Sch 42137 (**17**)	C_29_H_27_NO_10_ (549.5320)	*Actinoplanes* sp. SCC 1906	Antifungal	Cooper et al., 1992
Simaomicins α (**18**)*^b^*	C_28_H_25_NO_10_ (535.5050)	*Actinomadura madurae* subspecies *simaoensis*	Anti-gram-positive bacterial Cytotoxicity Antimalarial Anticoccidial	Lee et al., 1989; Maiese et al., 1990
Simaomicins β (**19**)	C_27_H_23_NO_10_ (521.4780)			
Sch 56036 (**20**)	C_30_H_31_NO_8_ (533.5770)	*Actinoplanes* sp. (SCC 2314, ATCC 55600)	Antifungal	Chu et al., 1998
Chrestoxanthone C (**21**)	C_26_H_23_NO_8_ (477.4690)	*Streptomyces chrestomyceticus* BCC 24770	Antifungal	Bunyapaiboonsri et al., 2016
Kibdeline A (**22**)	C_29_H_24_NO_10_Cl (581.9580)	*Kibdelosporangium* sp. MST-108465	Anti-gram-positive bacterial Cytotoxicity Nematocidal	Ratnayake et al., 2007; Sloman et al., 2011
Kibdeline B (**23**)	C_29_H_26_NO_10_Cl (583.9740)			
Kibdeline C (**24**)	C_29_H_28_NO_10_Cl (585.9900)			
Kibdeline A rhamnoside (**25**)	C_35_H_34_NO_14_Cl (728.1000)			
Kibdeline B rhamnoside (**26**)	C_35_H_36_NO_14_Cl (730.1160)			
Kibdeline C rhamnoside (**27**)	C_35_H_38_NO_14_Cl (732.1320)			
13-Oxokibdelone A (**28**)	C_29_H_22_NO_10_Cl (579.9420)			
25-Methoxy-24-oxokibdelone C (**29**)	C_30_H_31_NO_12_ (597.5730)			
25-Hydroxy-24-oxokibdelone C (**30**)	C_29_H_29_NO_12_ (583.5460)			
Isokibdelone A (**31**)	C_29_H_24_NO_10_Cl (581.9580)	*Kibdelosporangium* sp. MST-108465		Ratnayake et al., 2006
Isokibdelone B (**32**)	C_29_H_26_NO_10_Cl (583.9740)			
Isokibdelone C (**33**)	C_29_H_28_NO_10_Cl (585.9900)			
Isokibdeline A rhamnoside (**34**)	C_35_H_34_NO_14_Cl (728.1000)			

**Aromatic xanthones**				

Lysolipin I (**35**)*^a,b^*	C_29_H_24_NO_11_Cl (597.9570)	*Streptomyces violaceoniger* Tü 96	Anti-gram-positive bacterial Gram-negative bacteria *(Xanthomonas citri*)	Drautz et al., 1975; Rodrigues et al., 2018
Lysolipin X (36)*^b^*	C_29_H_26_NO_12_Cl (615.9720)			
Lysoquinone-TH1	C_25_H_18_O_9_ (462.4100)	*Streptomyces tendae* Tü 4042 (minimal PKS *llpD-F* and cyclase genes *llpCI-CIII*)	An inhibitor of PDE4	Hofeditz et al., 2018
Xantholipin (37)*^a^*	C_27_H_18_NO_10_Cl (551.8880)	*Streptomyces* sp.	Anti-gram-positive bacterial Cytotoxicity Inhibiting collagen production	Terui et al., 2003; Chen et al., 2011
Bromoxantholipin (38)	C_27_H_18_NO_10_Br (596.3420)	*Streptomyces flavogriseus* SIIA-A02191	Anti-gram-positive bacterial	Chen et al., 2011
Xantholipin B (39)	C_27_H_18_NO_9_Cl (535.8890)	*Streptomyces flocculus* CGMCC 4.1223	Anti-gram-positive bacterial Antifungal (*Candida albicans*) Cytotoxicity	Wu et al., 2017
15R-17,18-Dehydroxantholipin (40)	C_27_H_16_NO_9_Cl (533.8730)	*Streptomyces qinglanensis* 172205	Anti-gram-positive bacterial (*Staphylococcus aureus*) Antifungal (*Candida albicans*)	Xu et al., 2020
IB-00208 (41)	C_36_H_34_O_14_ (690.6540)	*Actinomadura* sp.	Anti-gram-positive bacterial Cytotoxicity	Rodriguez et al., 2003
Cervinomycin A_1_ (46)	C_29_H_23_NO_9_ (529.5010)	*Streptomyces cervinus* AM-5344	Anti-gram-positive bacterial, especially anti-anaerobic bacteria	Ömura et al., 1982, 1986; Nakagawa et al., 1987
Cervinomycin A_2_ (47)	C_29_H_21_NO_9_ (527.4850)			
Cervinomycin B_1_ (48)	C_29_H_25_NO_9_ (531.5170)	*Streptomyces* sp. CPCC 204980	Anti-gram-positive bacterial Cytotoxicity	Hu et al., 2019, 2020
Cervinomycin B_2_ (49)	C_29_H_23_NO_9_ (529.5010)			
Cervinomycin B_3_ (50)	C_29_H_23_NO_9_ (517.4900)			
Cervinomycin B_4_ (51)	C_29_H_21_NO_9_ (515.4740)			
Cervinomycin C_1_ (42)	C_28_H_23_NO_9_ (517.4900)			
Cervinomycin C_2_ (43)	C_28_H_21_NO_9_ (515.4740)			
Cervinomycin C_3_ (44)	C_26_H_19_NO_8_ (473.4370)			
Cervinomycin C_4_ (45)	C_26_H_17_NO_8_ (471.4210)			
Citreamicin α (52)*^b^*	C_36_H_31_NO_12_ (669.6390)	*Micromonospora citrea*	Against a range of gram-positive aerobic and anaerobic bacteria	Maiese et al., 1989; Carter et al., 1990
Citreamicin β (53)	C_35_H_29_NO_12_ (655.6120)			
Citreamicin γ (54)	C_33_H_25_NO_12_ (627.5580)			
Citreamicin ζ (55)	C_35_H_29_NO_12_ (655.6120)			
Citreamicin η (56)	C_31_H_23_NO_11_ (585.5210)			
Citreamicin δ (57)	C_30_H_21_NO_11_ (575.5260)	*Streptomyces vinaceus Streptomyces caelestis* Aw99c	Anti-gram-positive bacterial	Hopp et al., 2008; Liu et al., 2019
Citreamicin ε (58)	C_30_H_25_NO_11_ (575.5260)		Anti-gram-positive bacterial Antifungal (*Magnaporthe grisea*)	
Arixanthomycin A (59)*^a^*	C_37_H_37_NO_14_ (719.6960)	Environmental DNA	Anti-gram-positive bacterial Cytotoxicity	Kang and Brady, 2014
Arixanthomycin B (60)	C_29_H_23_NO_10_ (545.5000)			
Arixanthomycin C (61)	C_28_H_21_NO_10_ (531.4730)			
Neocitreamicin I (62)	C_36_H_31_NO_11_ (653.6400)	*Nocardia* sp. G0655	Anti-gram-positive bacterial	Peoples et al., 2008
Neocitreamicin II (63)	C_44_H_43_NO_15_ (825.8200)			

**Xanthones with sugar residues**				

Kigamicin A (64)	C_34_H_35_NO_13_ (665.6480)	*Amicolatopsis* sp. ML630-mF1	Anti-gram-positive bacterial Cytotoxicity	Kunimoto et al., 2003a,b; Someno et al., 2005
Kigamicin B (65)	C_40_H_45_NO_15_ (779.7920)			
Kigamicin C (66)	C_41_H_47_NO_16_ (809.8180)			
Kigamicin D (67)	C_48_H_59_NO_19_ (953.9880)			
Kigamicin E (68)	C_55_H_71_NO_22_ (1098.1580)			
FD-594 (69)*^a,b^*	C_47_H_56_O_20_ (940.9450)	Streptomyces sp. TA-0256	Anti-gram-positive bacterial Cytotoxicity	Kondo et al., 1998; Eguchi et al., 1999
MS 901809 (70)	C_47_H_56_O_20_ (940.9450)			
BE-13793X (71)	C_47_H_56_O_20_ (940.9450)			

*^a^The biosynthetic pathways of compounds 1, 35, 37, 59, and 69 have been studied by heterologous expression, enzymatic reaction, gene deletion, and/or bioinformatics analysis.*

*^b^The biosynthesis of compounds 18, 35, 36, 52, and 69 have been studied by isotope-labeled precursor feeding experiments.*

### Tetrahydroxanthones

**FIGURE 2:**
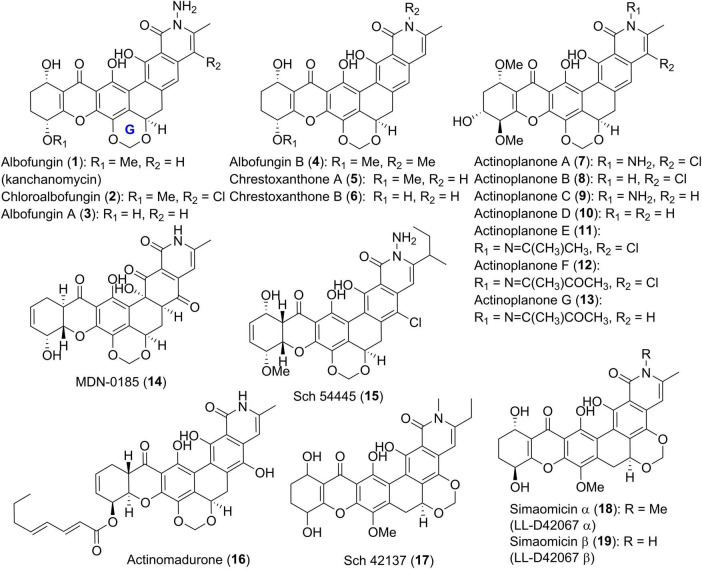
Chemical structures of compounds 1–19.

Since the identification of the first group member in 1972, albofungin (1, also known as kanchanomycin, Figure 2) with a unique tetrahydroxanthone-containg heptacyclic ring system from an *Actinomyces* species (Gurevich et al., 1972), polycyclic xanthone derivatives with intriguing structures and diverse bioactivities have attracted a lot of attentions. Recently, the structures of 1 and its chlorinated analogue chloroalbofungin (2), especially the methylene dioxybridge motif between C-17 and C-19 presented in albofungins, were unambiguously confirmed by single-crystal X-ray diffraction (Ye et al., 2020). Compounds 1 and 2, along with two new members albofungins A and B (3 and 4), were also uncovered from a *Streptomyces* species, *Streptomyces chrestomyceticus* (She et al., 2021). Two further albofungin derivatives, chrestoxanthones A and B (5 and 6) without substituents on the amide nitrogen, were found to be biosynthesized by the actinomycete *S. chrestomyceticus* BCC 24770 (Bunyapaiboonsri et al., 2016).

In 1988, Kobayashi et al. described the identification of a series of albofungin derivatives, actinoplanones A-G (7–13, Figure 2) isolated from the culture of *Actinoplanes* sp. R-304 (Kobayashi et al., 1988a,b). Their absolute configurations were determined from their ECD spectra and the Mosher’s method using chiral MTPA derivatives (Kobayashi et al., 1988a). MDN-0185 (14), and Sch 54445 (15) as tetrahydroxanthone derivatives with chiral centers at C-9 and C-14, were produced by *Micromonospora* sp. CA-256353 (Annang et al., 2018), and *Actinoplanes* sp. ATCC 55600 (Chu et al., 1997), respectively. Chemical investigation on *Actinomadura* sp. BCC 35430 led to the discovery of a yellow-brown polycyclic analogue, actinomadurone (16) with an unusual lipid chain at C-13 (Bunyapaiboonsri et al., 2017). Between 1989 and 1992, Sch 42137 (17) and simaomicins α and β (LL-D42067α and β, 18 and 19), three polycyclic tetrahydroxanthone compounds with a unique methylene dioxybridge between C-20 and C-22, were reported (Lee et al., 1989; Maiese et al., 1990; Cooper et al., 1992). The latter two compounds 18 and 19 from *Actinomadura madurae* subspecies *simaoensis*, were elucidated by NMR data and X-ray diffraction analysis (Lee et al., 1989; Maiese et al., 1990).

**FIGURE 3:**
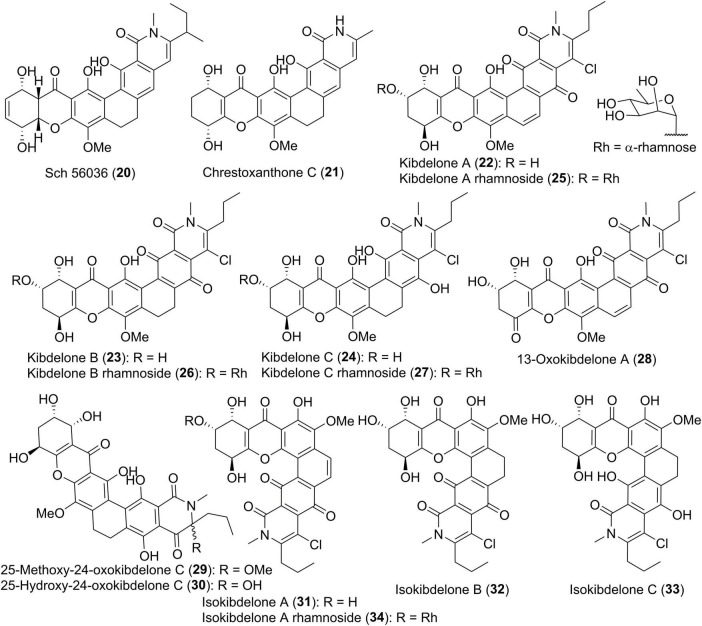
Chemical structures of compounds 20–34.

In comparison with albofungin-type compounds, the following polycyclic tetrahydroxanthones don’t possess the methylene dioxybridge motif, as exemplified by Sch 56036 (20, Figure 3; Chu et al., 1998), together with chrestoxanthone C (21) and kibdelones/isokibdelones (22–34) with a tetrahydroxanthone core (Ratnayake et al., 2006, 2007; Bunyapaiboonsri et al., 2016). It also has to be noted that most of them have a short chain at C-25 instead of the methyl in albofungins. Kibdelones including kibdelines A-C (22–24), kibdeline A-C rhamnosides (25–27), 13-oxokibdelone A (28), 25-methoxy-24-oxokibdelone C (29), and 25-hydroxy-24-oxokibdelone C (30), were discovered by Capon group in 2007 from a soil-derived actinomycete, *Kibdelosporangium* sp. MST-108465 (Ratnayake et al., 2007). Their absolute stereochemistry remained unclear until the total synthesis of compound 24 in 2011 (Sloman et al., 2011). Among kibdelones, 29 and 30 could be artifacts formed in methanol solutions during isolation and analysis process. Further chemical investigation on *Kibdelosporangium* sp. MST-108465 grown on wheat culture instead of barley yielded four isokibdelones, isokibdelones A-C (31–33) and isokibdeline A rhamnoside (34), with an unprecedented heterocyclic twist skeleton (Ratnayake et al., 2006). Notably, a chemical equilibrium was observed in kibdelones, which was responsible for the mixtures of 22–24, 25–27, or 31–33 under mild conditions in MeOH (Ratnayake et al., 2006, 2007). The underlying equilibrium mechanism was proposed to involve oxidation, hydroquinone/quinone interconversions, as well as keto/enol tautomerizations (Ratnayake et al., 2007).

### Aromatic Xanthones

**FIGURE 4:**
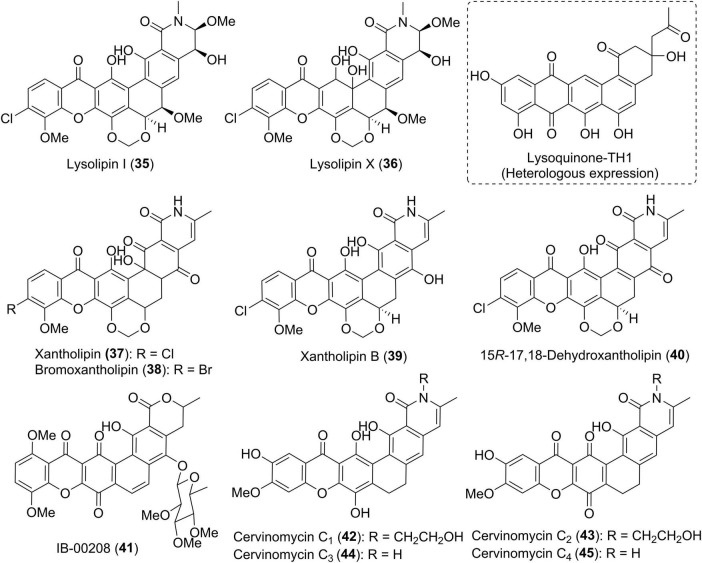
Chemical structures of compounds 35–45.

Fully aromatic xanthone derivatives typically possess the polycyclic framework fused to a methylene dioxybridge or a fused oxazolidine ring. The xanthone ring is sometimes halogenated by chlorine or bromine. Lysolipins I and X (35 and 36) (Figure 4) are representative chlorinated xanthone polyketides that were firstly isolated from *Streptomyces violaceoniger* Tü 96 in 1975 (Drautz et al., 1975; Winter et al., 2013). Compound 36 was found to be unstable and could be converted into 35. Terui et al. (2003) isolated a lysolipin analogue xantholipin (37) from a *Streptomyces* species, whose absolute configuration was assigned by the chemical modification and Mosher’s method. Its bromo analogue, bromoxantholipin (38), was uncovered in 2011 from *Streptomyces flavogriseus* SIIA-A02191 (Chen et al., 2011). The inactivation of an aminotransferase StnR in the streptonigrin biosynthesis in *Streptomyces flocculus* CGMCC 4.1223 generated a mutant strain WJN-1, which was found to produce xantholipin B (39) (Wu et al., 2017). 15R-17,18-dehydroxantholipin (40) was isolated as a red solid from the mangrove-derived *Streptomyces qinglanensis* 172205 in which the highly expressed enterocin biosynthetic pathway was deleted (Xu et al., 2020).

**FIGURE 5:**
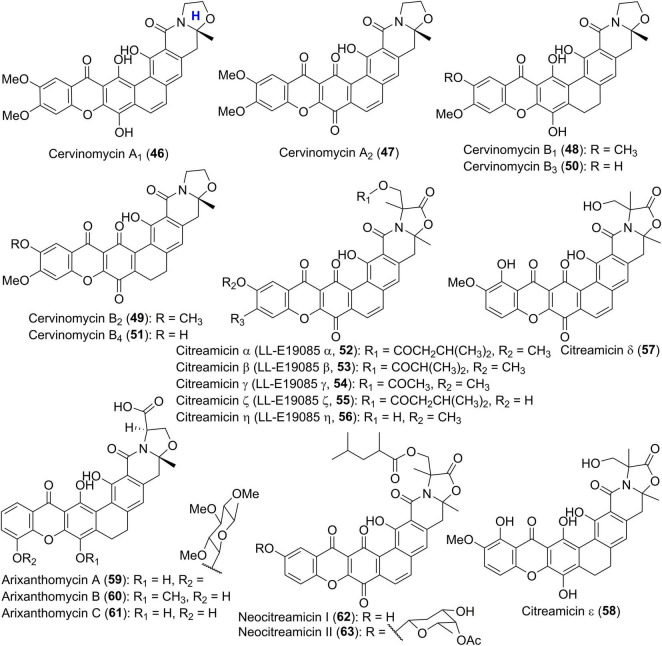
Chemical structures of compounds 46–63.

IB-00208 (41) and cervinomycins (42–51) (Figures 4, 5) are aromatic xanthone-containing polycyclic secondary metabolites without a methylene dioxybridge unit. Among them, compound 41 with a isochromane substructure rather than the isoquinone along with a sugar moiety, was obtained from *Actinomadura* sp. (Rodriguez et al., 2003). Early in 1982, cervinomycin A_1_ (46), and its quinone derivative cervinomycin A_2_ (47) were obtained from the liquid culture of a soil-derived *Streptomyces cervinus* AM-5344 by Ömura group (Ömura et al., 1982). The physico-chemical properties and NMR investigation on methylated or acetylated derivatives of 46 and 57 (Ömura et al., 1986; Nakagawa et al., 1987), completed the structural identification and indicated the presence of an additional oxazolidine ring fused to the isoquinone. Recently, Wu and co-workers were interested in the bioactive secondary metabolites from *Streptomyces* sp. CPCC 204980 which was isolated from a soil sample (Hu et al., 2019, 2020). Two types of cervinomycin derivatives, cervinomycins B_1–4_ (48–51) (Hu et al., 2019), and cervinomycins C_1–4_ (42–45) (Hu et al., 2020), were identified by comprehensive analyses of MS, NMR, as well as X-ray diffraction data. In comparison with 46 and 57, 48–51 possessed a hydro-D ring while 42–45 featured an open or loss of A ring in the polycyclic framework (Hu et al., 2019, 2020).

Citreamicins α, β, γ, ζ, and η (52–56) (Figure 5), previously designated as LL-E19085 antibiotics, were structurally related to cervinomycins, and were produced by an actinomycete strain, *Marinirhabdus citrea* (Maiese et al., 1989; Carter et al., 1990). Antimicrobial activity guided isolation led to the discovery of two new members citreamicins δ and ε (57 and 58) (Hopp et al., 2008; Liu et al., 2019). Based on the soil-derived DNA libraries, Kang and Brady applied the ketosynthase beta (KS_β_) sequence as phylogenetic marker to find an interesting gene cluster *ARX* of which KS_β_ sequence AZ33 exhibited a new branch compared with known KS_β_ gene sequences (Kang and Brady, 2014). Following this genome mining approach, three novel pentangular polyphenols arixanthomycins A-C (59–61) were uncovered (Kang and Brady, 2014). Chemical investigation of a soil-derived *Nocardia* stain G0655 led to the isolation of two citreamicin congeners, neocitreamicins I and II (62 and 63), while their stereochemistry was not determined (Peoples et al., 2008).

### Xanthones With Sugar Residues

**FIGURE 6:**
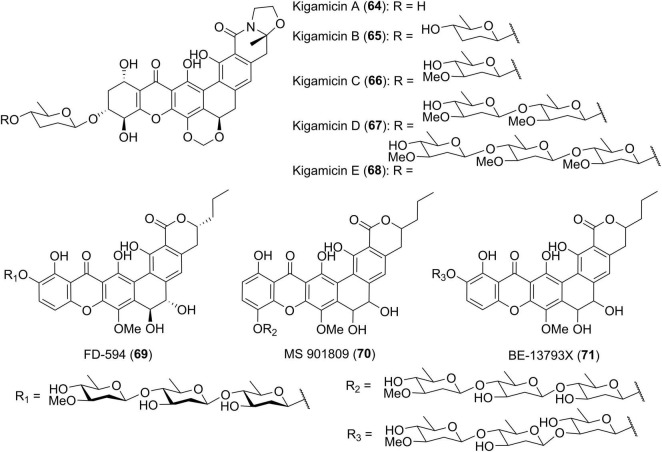
Chemical structures of compounds 64–71.

Diverse sugar residues are often found in polycyclic xanthones and are typically attached to the A ring of the xanthone. Several examples have been previously indicated by compounds 25–27, 34, 41, 59, and 63. In the course of discovering new antitumor antibiotics from *Amicolatopsis* sp. ML630-mF1, scientists from Japan isolated five yellow pigments, kigamicins A-E (64–68) (Figure 6; Kunimoto et al., 2003a,b). This kind of kigamicins was composed of an octacyclic ring system that was further attached by mono-, di-, tri- or tetrasaccharide moieties. Their stereochemistry including the absolute configuration of amicetose or oleandrose moieties was unambiguously determined by a combined analysis of X-ray crystallographic analysis and chemical degradation (Someno et al., 2005). FD-594 (69), MS 901809 (70), and BE-13793X (71) were structurally related xanthones which were attached with a trisaccharide including a D-oleandrose and two D-olivoses (Kondo et al., 1998). The stereochemistry of compound 69 including its rare solvent-dependent atropisomeric phenomenon was established based on single crystal X-ray diffraction as well as CD and NMR data (Eguchi et al., 1999).

## Biosynthesis

Polycyclic xanthones have attracted a lot of biosynthetic studies due to their angular fused hexacyclic framework. Particularly intriguing is the formation of the characteristic xanthone, the isoquinone, and the methylenedioxy bridge (Winter et al., 2013). Isotope-labeled precursor feeding experiments proved that polycyclic xanthone derivatives are assembled *via* a type II minimal PKS-derived single polyketide chain (Carter et al., 1989, 1991; Bockholt et al., 1994; Kondo et al., 1998). This minimal PKS consists of three monofunctional enzymes including the ketosynthase α (KS_α_), ketosynthase β/chain-length factor (KS_β_/CLF), and acyl carrier protein (ACP) (Zhang et al., 2012). The single polyketide chain is subsequently cyclized and aromatized by diverse enzymes, following by various post-PKS modifications, such as oxygenations, rearrangements, halogenations, methylations, and/or glycosylations, to afford a number of polycyclic xanthones (Zhang et al., 2012; Kang and Brady, 2014).

**FIGURE 7:**
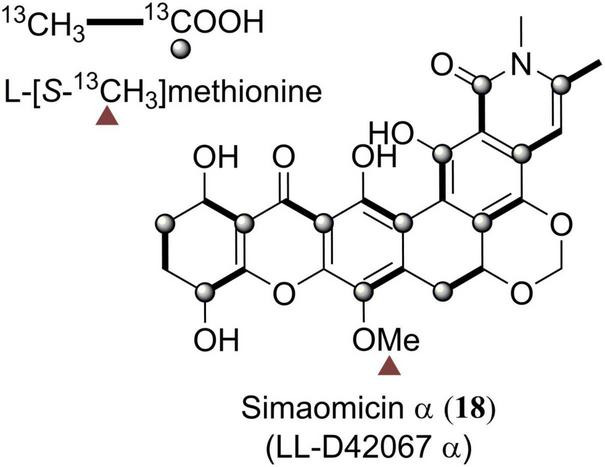
Biosynthetic origins of carbons in simaomicin α (18).

Simaomicin α (18) was firstly subjected to biosynthetic studies (Figure 7). By feeding ^13^C-labeled methionine and acetates into the culture of simaomicin α-producing strain and based on the ^13^C NMR spectroscopic analysis of the enriched carbon signals, it was proven that compound 18 was derived from a polyketide chain (Figure 7; Carter et al., 1989). Similar isotope-labeled precursor feeding experiments were also carried out for 35 (Bockholt et al., 1994), 36 (Bockholt et al., 1994), 52 (Carter et al., 1991), and 69 (Kondo et al., 1998), indicating that they are all originated from a single polyacetate precursor.

**FIGURE 8:**
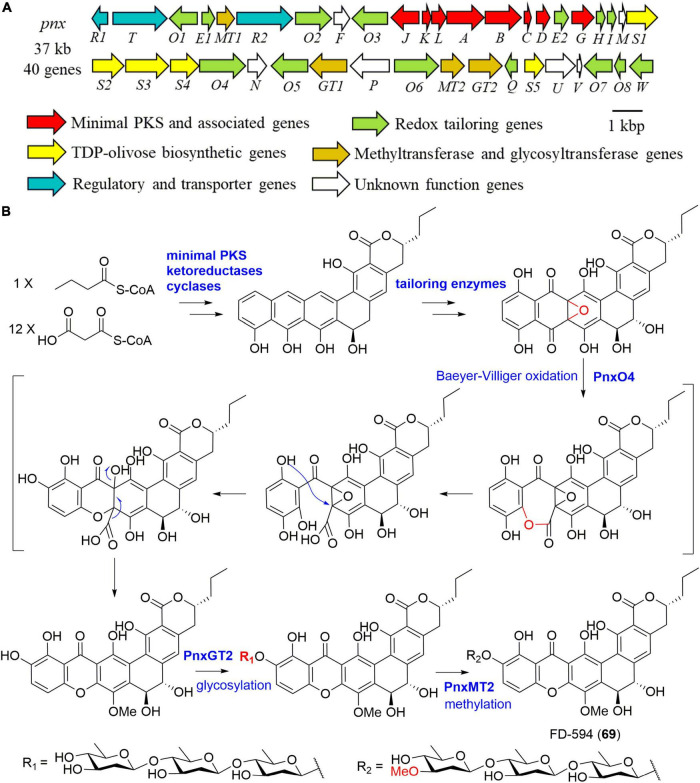
The biosynthesis of FD-594 (69). (A) The gene cluster *(pnx*) of compound 69; (B) the proposed biosynthetic pathway of compound 69.

Further clone studies on the BGC of lysolipins from *Streptomyces tendae* Tü 4042 were performed in 2010 (Lopez et al., 2010). The BGC was proposed to include a lot of genes encoding proteins responsible for redox tailoring steps in addition to genes coding for a minimal PKS, cyclases, methyltransferases, a halogenase, an amidotransgerase, and regulatory enzymes (Lopez et al., 2010). Heterologous expression of the minimal PKS of lysolipin I (35) in combination with its cyclic genes in *S. albus* J1074 afforded a polyketide lysoquinone-TH1 that has an intact aromatic polycyclic system (Hofeditz et al., 2018). Kudo et al. (2011) cloned the 37 kb BGC (*pnx*) of compound FD-594 (69) from the producer *Streptomyces* sp. TA-0256 (Figure 8A). A putative flavin adenine dinucleotide (FAD)-dependent monooxygenase PnxO4 was presumed to catalyze the key Baeyer-Villiger oxidation-mediated ring opening process, leading to the construction of the unique xanthone substructure (Figure 8B). Moreover, a glycosyltransferase, PnxGT2 for catalyzing the triple olivose transfers, and a methyltransferase, PnxMT2 for completing the methylation of the triolivoside, were characterized enzymatically *in vitro* (Figure 8; Kudo et al., 2011).

**FIGURE 9:**
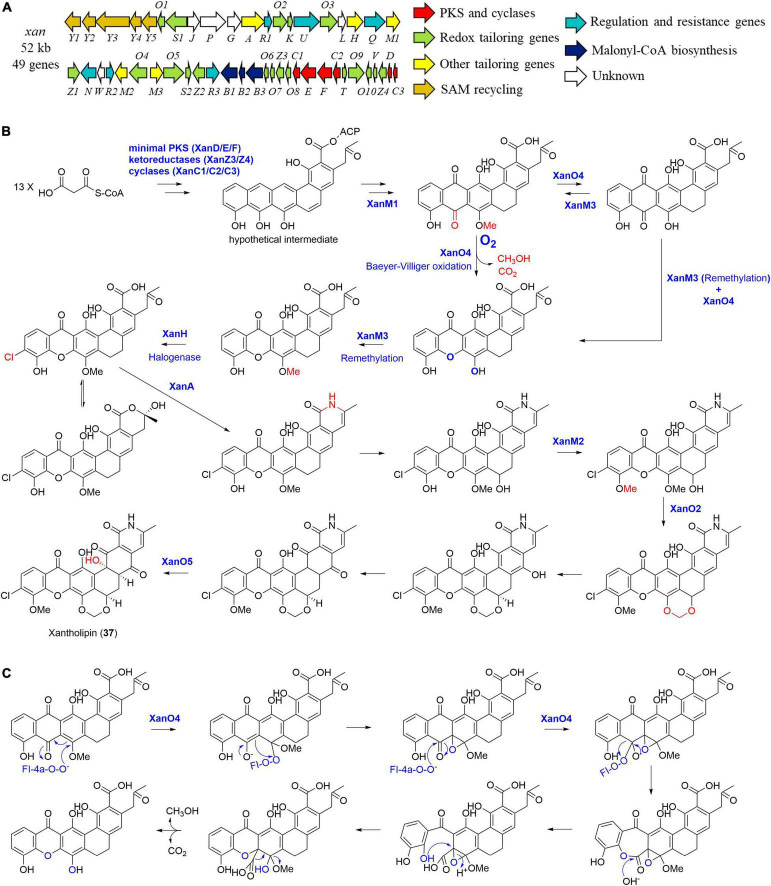
The biosynthesis of xantholipin (37). (A) The gene cluster *(xan*) of compound 37; (B) the proposed biosynthetic pathway of compound 37; (C) the possible enzymatic mechanism of the multifunctional monooxygenase XanO4.

In 2012, the entire 52 kb xantholipin (37) BGC (*xan*) from *S. flavogriseus* was cloned and sequenced by You and co-workers (Figure 9A; Zhang et al., 2012). Further individual gene deletion experiments identified four tailoring enzymes including a multifunctional monooxygenase XanO4 for the xanthone scaffold *via* a Baeyer-Villiger oxidation, an amide synthetase XanA for catalyzing the amide bond, a P450 monooxygenase XanO2 for methylene dioxybridge formation, as well as a monooxygenase XanO5 for the hydroxylation of the carbon backbone at C-4 (Figure 9B; Zhang et al., 2012). Three paralogous methyltransferases were also found in the BGC. When XanM1 was deleted, there were no observed product nor intermediate, enabling You group to propose that XanM1 acted on a hypothetical ACP-binding intermediate (Zhang et al., 2012; Kong et al., 2020b). More intriguingly, purified XanM1 could also make the substrates of XanM2 and XanM3 to be methylated. In addition, three methyltransferases XanM1-M3 showed minor functional overlaps with similar methylation activities toward several intermediates although they possessed highly substrate-dependent regioselectivity. As further indicated by phylogenetic analysis and ancestral sequence reconstruction, XanM1-M3 were finally proposed to be diversified from a common ancestor (Kong et al., 2020b).

More importantly, the XanO4-mediated Baeyer-Villiger reaction from anthraquinone to xanthone is accompanied by an indispensable cryptic demethoxylation, which is proven to be general in polycyclic xanthones (Figure 9C; Kong et al., 2016). This unique mechanism of XanO4 perhaps initiates the oxidation at C-17, contributing to the epoxide intermediate formation (Figure 9C). This breaks the ring aromaticity and stimulates the subsequent Baeyer-Villiger oxidation reaction, followed by decarboxylation and cleavage of C-17 methoxy group to yield the xanthone ring (Kong et al., 2016). When the xanthone was constructed, the methyltransferase XanM3 enabled its remethylation, followed by halogenation catalyzed by an indispensable FAD-dependent halogenase XanH (Kong et al., 2020a). XanH was found to accept the freely diffusing substrate with an angular polycyclic aromatic scaffold, which was different from those of the exclusively studied FAD-dependent halogenases (Kong et al., 2020a).

**FIGURE 10:**
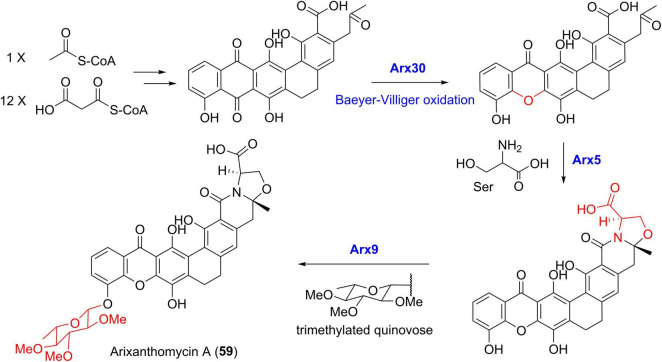
The proposed biosynthetic pathway of arixanthomycin A (59).

For the biosynthesis of arixanthomycin A (59) (Figure 10), the Baeyer-Villiger oxidation was predicted to be catalyzed by Arx30 with 61% identity to XanO4 (Kang and Brady, 2014). Further serine incorporation into the terminal carboxyl group following by the oxazolidine ring construction was possibly catalyzed by the amidotransferase Arx5. The final addition of the trimethylated quinovose sugar unit was achieved by the putative glycosyltransferase Arx9 (Kang and Brady, 2014). Recent genome sequencing revealed the presence of a 72 kb albofungin (1) gene cluster (*alb*) in *S. chrestomyceticus* (She et al., 2021), which was confirmed by heterologous expression of *alb* in *S. coelicolor*. In addition, the compound 1 biosynthetic pathway exhibited high similarity with those of lysolipins and xantholipins (She et al., 2021).

## Biological Activity

Polycyclic xanthones have aroused considerable interest of chemists and biologists since most members displayed selective inhibitory activity against gram-positive bacteria at nanomolar concentrations but displayed moderate to no efficiency against gram-negative bacteria (Winter et al., 2013). Gram-positive methicillin-resistant *Staphylococcus aureus* (MRSA), and vancomycin-resistant *Enterococcus* (VRE) strains were also observed to be strongly disrupted by this class of natural products (Peoples et al., 2008; Wu et al., 2017; Hu et al., 2019; She et al., 2021). Several tetrahydroxanthone-containing molecules with chiral centers at C-9 and C-14 tended to have remarkable antifungal activities (Chu et al., 1997, 1998). Also particularly intriguing is the significant cytotoxic activities of polycyclic xanthones with IC_50_ values in the nanomolar range, and this class of compounds are often recognized as anticancer antibiotics (Winter et al., 2013; She et al., 2021). There have also been some works reporting activities ranging from potent insecticidal activities including antimalarial, nematocidal, and anticoccidial actions to protein inhibition effects (Maiese et al., 1990; Ratnayake et al., 2007; Ui et al., 2007; Hofeditz et al., 2018). The structure-activity relationship of this compound group is becoming clear after extensive literature review.

### Antimicrobial Activities

This family of natural products initially attracted interest due to their antimicrobial activity. The first member of this family albofungin (1), together with its analogues 2–4 showed significant antibacterial activities at a nanomolar range against gram-positive bacteria including MASA (ATCC 43300), *S. aureus* (ATCC 25923 and B04), and *Bacillus subtilis* ZK31 (She et al., 2021). In comparison with 4, more efficient antibacterial activity of 1-3 was observed, suggesting that the *N*-aminoamide unit was crucial. Interestingly, compound 3 also possessed most potent activities toward tested gram-negative bacteria at a low micromolar range (She et al., 2021), indicating that its hydroxyl group in the A ring might be important toward penetrating the outer membrane of gram-negative bacteria. Fully aromatic xanthone-containing 35 also showed significant inhibitory activity against both gram-positive and gram-negative bacteria in a nM-range (Hofeditz et al., 2018; Rodrigues et al., 2018). In contrast, its polycyclic biosynthetic precursor lysoquinone-TH1 without the xanthone motif showed weak activity against *Staphylococcus lentus*, *Staphylococcus epidermidis*, and *Propionibacterium acnes* at 100 μM (Hofeditz et al., 2018). These results suggest that the xanthone core featured in polycyclic xanthone derivatives might be important for antimicrobial bioactivity.

Biological assay *in vitro* revealed the antifungal potential of compounds 5–13 (Kobayashi et al., 1988b; Bunyapaiboonsri et al., 2016). More particularly, compounds 7–13 inhibited spore germination of the rice blast fungus *Pyricularia oryzae* with IC_50_ values in the range of 0.0016–0.106 μg/mL (Kobayashi et al., 1988b). Moreover, 7 not only demonstrated significant activity against gram-positive bacteria (MIC < 0.0007 μg/mL), but also was moderately effective against gram-negative bacteria (MIC 0.05–12.5 μg/mL) (Kobayashi et al., 1988b). As tetrahydroxanthone antibiotics with chiral centers at C-9 and C-14, Sch 54445 (15) and Sch 56036 (20) were demonstrated to be broad-spectrum antifungal agents against diverse yeasts and dermatophytes, such as *Candida albicans* with an MIC value less than 2 nM (Chu et al., 1997, 1998). Compound 15 was also found to had better antifungal efficiency than other tetrahydroxanthone derivatives 1 and 17 which do not possess the stereo genic centers at C-9 an C-14 and the chlorine substitution on the E ring (Chu et al., 1997). Among them, 17 showed the weakest antifungal activities. Compound 18 with similar structural features as those of 17 having the methylene dioxybridge unit located between C-20 and C-22, also had weak or no activities against tested fungi although it selectively inhibited gram-positive bacteria with MIC values equal to or less than 0.06 μg/mL (Maiese et al., 1990). In 2017, Bunyapaiboonsri et al. showed that the structurally specific tetrahydroxanthone-containing 16 at the concentrations of 0.64–5.10 μM strongly inhibited four plant pathogens including *Colletotrichum capsici*, *Colletotrichum gloeosporioides*, *Curvularia lunata*, and *Alternaria brassicicola* (Bunyapaiboonsri et al., 2017). The above combined results indicate that several key structural features presented in polycyclic xanthones, such as the tetrahydroxanthones with chiral centers at C-9 and C-14, and the methylene dioxybridge unit between C-17 and C-19, could play an important role in antifungal activity.

Three congeners 22–24 were found to be 10 fold more potent against the gram-positive *B. subtilis* than 1, and exhibited weak or no effect toward the gram-negative *Escherichia coli* or fungus *C. albicans* (Ratnayake et al., 2007). Similarly, Chen et al. tested the antibacterial activities of 37 and 38 (Chen et al., 2011), which displayed close potency against gram-positive bacteria including MRSA SIA 98839 with an MIC value of 0.25 μg/mL, but poor efficiency against gram-negative bacteria. Similar biological characteristics were also observed for compound 39 (Wu et al., 2017). Particularly, 39 demonstrated significant activity against MRSA Mu50 with an MIC value of 0.025 μg/mL. In addition, 39 was more effective than 37 against *C. albicans* (0.31 μg/mL, 4-fold) and *Candida sake* (0.08 μg/mL, 4-fold), indicating the crucial role of aromatic E-ring in bioactivity (Wu et al., 2017). Two further fully aromatic xanthones, 40 and 41, were also potent selective antimicrobial agents for gram-positive bacteria in a nanomolar range (Malet-Cascon et al., 2003; Xu et al., 2020). Therefore, polycyclic xanthones seem to have selective anti-gram-positive activities.

The selective antibacterial activity against gram-positive bacteria was further supported by the structures and activities of 52–56, and 59–69. Citreamicins 52–56 from *M. citrea* were effective *in vitro* against a range of gram-positive aerobic and anaerobic bacteria, but were noticeably less active against the selected gram-positive anaerobic *Bacteroides fragilis* (Maiese et al., 1989; Carter et al., 1990). Among them, compound 56 was the most potent one against several gram-positive strains with MIC values of less than 0.015 μg/mL (Carter et al., 1990). As expected for aromatic xanthones (59–61) at the concentration 50 μg/mL were inactive in inhibiting the fungus *Saccharomyces cerevisiae*, and gram-negative bacterium *E. coli* DRC39 (Kang and Brady, 2014). On the other hand, 59 was the most biologically active one against three gram-positive bacteria MRSA USA300, *B. subtilis* RM125, and VRE EF16, bringing out the sugar role for antibacterial activity. The antibacterial activities of neocitreamicins I and II (62 and 63) were determined by serial dilution method in liquid media (Peoples et al., 2008). Both compounds displayed good antibacterial activity against selected three MRSA and two VRE strains with MIC values of 0.06–0.50 μg/mL (Peoples et al., 2008). Other polycyclic xanthones with polysaccharide moieties (64–69) also selectively inhibited the growth of gram-positive bacteria (Qiao et al., 1998; Kunimoto et al., 2003a).

In addition to have strong selective activity against gram-positive bacteria, polycyclic xanthones, for example the above mentioned citreamicins, were also potent anti-anaerobic agents. Cervinomycins A_1_ and A_2_ (46 and 47) were also reported to be highly active against gram-positive and gram-negative anaerobic bacteria, such as *Eubacterium lentum*, *Bifidobacterium bifidum*, *Clostridium perfringens*, *Lactobacillus acidofilus*, *Peptococcus prevotii*, and *B. fragilis* with MIC values ranging from 0.006 to 0.195 μg/mL (Ömura et al., 1982). Acetyl modifications of 46 and 47 were found to enhance their anti-anaerobic activity as well as solubility (Nakagawa et al., 1986, 1987). The mode of action study on an acetyl derivative of 46, triacetylcervinomycin A_1_ with high solubility and low toxicity, indicated that this kind of compound might interact with phospholipids in the bacterial cytoplasmic membrane (Tanaka et al., 1989). Cervinomycins B_1_-B_4_ (48–51) displayed promising activity against MRSA and VRE, and were inactive against gram-negative bacteria (Hu et al., 2019). The dihydro-D ring should be important for antibacterial activity as indicated by the enhanced activity of 49 (MIC 0.06–0.12 μg/mL) compared with 47 (MIC 0.12–1.0 μg/mL). On the other hand, the 11-*O*-methyl group could reduce antibacterial activity, which was revealed by the IC_50_ values of 48 (0.03–0.12 μg/mL) and 50 (0.008–0.03 μg/mL) (Hu et al., 2019). Similarly, cervinomycins C_1_-C_4_ (42–45) with the loss of oxazolidine ring compared with 46–51, also showed comparable anti-gram-positive bacterial activity and were inactive against gram-negative bacteria (Hu et al., 2020). Therefore, the contribution of the G ring of these cervinomycins seems to be not important for antimicrobial activity.

### Cytotoxicity

Polycyclic xanthones are judged to be important antitumor natural products and are broadly active toward various cancer cells in the nanomolar to low micromolar range. Albofungins (1–4) exhibited strong antitumor activities toward MCF-7, Hela, and HepG2 cell lines with IC_50_ values of 0.003 to 0.9 μM (She et al., 2021). Among them, 3 with the strongest cytotoxicity triggered cell apoptosis in MCF-7 and Hela cells. In 1988, actinoplanones (7–13) were obtained as cytotoxic agents against HeLa cells. Particularly, compounds 7, 9, and 13 were extremely active toward HeLa cells with IC_50_ values less than 0.00004 μg/mL (Kobayashi et al., 1988a,b). Compound 7 was further evaluated its antitumor activities against 11 cell lines, showing 100 to 1000 fold stronger cytotoxicity than controls mitomycin C and doxorubicin. Strong cytotoxicity of 16 toward African green monkey kidney fibroblast (Vero) cells was observed, showing an IC_50_ value of 0.008 μM (Bunyapaiboonsri et al., 2017). Polycyclic xanthone 18 demonstrated promising antiproliferative activity against some cancer cell lines with IC_50_ values in the range of 0.3-19 nM (Koizumi et al., 2009), while 15 had *in vivo* toxicity in mice *via* intravenous injection with an LD_50_ value of 1 mg/kg (Chu et al., 1997).

Kibdelones A-C (22–24) obtained by Capon and co-workers, displayed selective cytotoxic properties against a variety of human cancer cell lines, such as SR tumor cell line and SN12C cell carcinoma with GI_50_ values less than 5 nM (Ratnayake et al., 2007). In contrast, three isomeric congeners, the isokibdelones A-C (31–33) didn’t display significant antitumor activity and their GI_50_ values were around 10 to 200 fold less potent against selected tumor cell lines (Ratnayake et al., 2006). These results indicated that the skeleton twist/connectivity patterns diverged in 22–24 and 31–33 pose great influence on their activity.

Xantholipin (37) and xantholipin B (39) were evaluated for their cytotoxicity against five human cancer cell lines A549, HL-60, KB, BGC-803, and MCF-7 (Wu et al., 2017). They possessed potent activities with IC_50_ values in the range from 0.0088 to 0.43 μg/mL and 39 revealed stronger cytotoxic effects than 37 (Wu et al., 2017). Another structural analogue 40 showed moderate cytotoxicity on HeLa and MCF-7 cells with IC_50_ values around 6.0 μM, and was 10-fold less active than that of the drug paclitaxel (Xu et al., 2020). In 2003, nanomolar concentrations of 41 were found to inhibit P388D1, A-549, HT-29, and SKMEL-28 cancer cell lines (Malet-Cascon et al., 2003).

Cervinomycins (42–51) not only displayed significant antibacterial activity, but also had potent cytotoxicity *in vivo* and *in vitro*. For example, the acute toxicity LD_50_ values of 46 and 47 in mice were the same value 50 mg/kg (Ömura et al., 1982). Compounds 48–51 were extremely cytotoxic to three cancer cell lines S1990, PC3, and H1299 *in vitro* (Hu et al., 2019). The cytotoxic IC_50_ values of 48–51 were obviously lower than those of 47, indicating that a dihydro-D ring prefers to enhance cytotoxicity (Hu et al., 2019). Specifically, compound 48 was the mostly active one and had IC_50_ values of 2.2, 2.2, and 0.1 nM against S1990, PC3, and H1299, respectively, in comparison with 703.9, 1312.0, and 252.3 nM for 47. Similarly, 42-45 showed potent selective cytotoxicity toward the human cell line HCT116 rather than BxPC-3, and their IC_50_ values were noticeably lower than those of the positive control doxorubicin (Hu et al., 2020).

Similar to the antibacterial characteristics, arixanthomycin A (59) with a sugar residue at C-13 showed particularly better antiproliferative activity than its derivatives 60 and 61, and had IC_50_ values of 0.15–0.83 μM against breast and colon cancer cell lines (Kang and Brady, 2014). This result is rather intriguing because most polycyclic xanthones possessed cytotoxicity without sugar moieties. As one antitumor member of kigamicins, compound 67 inhibited various mouse tumor cell lines at IC_50_ values of around 1 μg/mL (Kunimoto et al., 2003a). FD-594 (69) had comparable antiproliferative activity to adriamycin (Qiao et al., 1998). This kind of polycyclic xanthones, such as 18 and 46, might function by arresting the tumor cell cycle at G1 phase accompanied with apoptotic cell death (Koizumi et al., 2009).

### Insecticidal and Enzyme Inhibitory Activities

Regarding the insecticidal and enzyme inhibitory activities of polycyclic xanthones, only few reports can be collected, but still reveal the potency of this class of molecules. Compared with chloroquine, artemisinin, and artemether, simaomicin α (18) had remarkably stronger antimalarial activities against *Plasmodium falciparum* strains K1 (drug-resistant) and FCR3 (drug-sensitive) with IC_50_ values of 0.045 and 0.0097 ng/mL, respectively (Ui et al., 2007). It affected *P. falciparum* development in a time and concentration dependent manner (Ishiyama et al., 2008). More importantly, its cytotoxic activity against human diploid embryonic cell line MRC-5 (IC_50_ 4.0 ng/mL) was significantly weaker than its antimalarial activities (Ui et al., 2007). Furthermore, compound 18 also displayed *in vivo* anticoccidial activity against a spectrum of chicken coccidian and almost completely prevented lesions at dosage of 1 g/ton in the diet of chickens (Maiese et al., 1990). These results indicated that 18 is a potent lead compound for developing insecticidal agents, especially antimalarial drugs. MDN-0185 (14) was another reported antimalarial polycyclic xanthone with potency comparable to 18, and showed an IC_50_ of 9 nM against *P. falciparum* 3D7 (Annang et al., 2018). Polycyclic 22–24 displayed potent nematocidal activity against *Haemonchus contortus* and provided LD_99_ 0.67, 2.2, and 8.5 nM, respectively with albofungin (1) as the control (LD_99_ 1.2 nM) (Ratnayake et al., 2007).

As a biosynthetic precursor of polycyclic xanthone derivatives, lysoquinone-TH1 without the xanthone unit was found to inhibit phosphodiesterase 4 (PDE4) and had potential to treat PDE4-related chronic obstructive pulmonary diseases (Hofeditz et al., 2018). Intriguingly, it was 10-fold more potent than the clinical drug rolipram. As an inhibitor of HSP47 gene expression, xantholipin (37) potently inhibited collagen production with an IC_50_ value of 27 nM (Terui et al., 2003).

## Conclusion and Future Perspectives

A number of structurally novel and biologically active polycyclic xanthone derivatives are increasingly being described. Herein, we provide a comprehensive review on 71 polycyclic xanthones with a highly oxygenated, angular hexacyclic framework. They were discovered from diverse actinomycete genera, such as *Streptomyces*, *Actinoplanes*, *Actinomadura*, *Micromonospora*, and *Kibdelosporangium* species. The structural novelty and diversity of these molecules result from the biosynthetic capability of these fascinating producers to assemble and modify the type II PKS-derived single polyacetate chain, especially the intriguing enzymatic Baeyer-Villiger oxidation to form the xanthone ring. In addition, this class of compounds exhibit promising bioactivities, and frequently reported biological activities are antimicrobial and cytotoxic properties in the nanomolar range, making them as potent anticancer antibiotics.

Despite these noticeable successes in isolation, biosynthetic and biological studies on polycyclic xanthones, exploring this family of natural products as drug candidates is challenging. For the BGCs of polycyclic xanthones, numerous genes coding for a minimal PKS, cyclases, monooxygenases, methyltransferases, amidotransgerase(s), glycosyltransferases, and/or a halogenase are well-studied now. However, a lot of genes encoding proteins responsible for tailoring and regulatory steps remain elusive, necessitating synthetic biology approaches to identify their functions before engaging in the chemical investigation processes. In addition to the attracting anticancer antibiotic characteristics, discovery of more potent insecticidal agents from polycyclic xanthones, such as the analogues of simaomicin α (18), could be expected in the future. This needs more experimental efforts for confirming the insecticidal potential of this family of polycyclic xanthone derivatives. The structural and bioactivity data summarized in this review tentatively suggested that the fully aromatic-, or tetrahydroxanthone core is the potent pharmacophore, and structural modifications on the hexacyclic framework influence the activity. However, the structure-activity relationship and modes of action of this group of natural products remains unclear, and more chemical investigations accompanied with bioassay are really needed. A successful structure-activity summary requires collecting and exploring high-quality and accurate data in large quantities. Obtaining molecular targets for isolates can also be a challenging task based on the frequently used phenotypic assays as summarized in this review, and new advanced approaches for confirming modes of action are necessary.

To exploit more polycyclic xanthones from actinomycetes for drug discovery, recent advances in genome sequencing and omics-related technology with the aid of artificial intelligence tools have provided a great opportunity to connect cryptic biosynthetic pathways (BGCs) to novel chemical structures of polycyclic xanthones (Li et al., 2022). These biosynthetic pathways could come from the uncharacterized BGCs of cultivable actinomycetes and the untapped BGCs in uncultured actinomycetes/environmental samples (Kang and Brady, 2014; Niu, 2018), allowing the shift from the traditional new actinomycete species-based natural product discovery paradigm to a new genomics-driven compound discovery campaign. This is promising in the targeted identification of known polycyclic xanthones and their novel analogues, benefiting the discovery of novel drug leads.

## Author Contributions

H-QY wrote the manuscript. H-XL provided the manuscript idea. GL collected and reorganized the literature data. GL and H-XL supervised the research work and revised the manuscript. All authors reviewed the manuscript and approved the submitted version.

## Conflict of Interest

The authors declare that the research was conducted in the absence of any commercial or financial relationships that could be construed as a potential conflict of interest.

## Publisher’s Note

All claims expressed in this article are solely those of the authors and do not necessarily represent those of their affiliated organizations, or those of the publisher, the editors and the reviewers. Any product that may be evaluated in this article, or claim that may be made by its manufacturer, is not guaranteed or endorsed by the publisher.
